# Transcriptome profiling provides insights into dormancy release during cold storage of *Lilium pumilum*

**DOI:** 10.1186/s12864-018-4536-x

**Published:** 2018-03-14

**Authors:** Wang Wang, Xiaoxia Su, Zhongping Tian, Yu Liu, Yunwei Zhou, Miao He

**Affiliations:** Northeast Forestry University, Harbin, China

**Keywords:** Low temperature - *Lilium pumilum* - transcriptome - dormancy release

## Abstract

**Background:**

Bulbs of the ornamental flower *Lilium pumilum* enter a period of dormancy after flowering in spring, and require exposure to cold for a period of time in order to release dormancy. Previous studies focused mainly on anatomical, physiological and biochemical changes during dormancy release. There are no dormancy studies of the northern cold-hardy wild species of *Lilium* at the molecular level. This study observed bulb cell and starch granule ultrastructures during cold storage; and analysed the transcriptome using sequencing. The combination of morphological and transcriptomic methods provides valuable insights into dormancy release during cold storage of *Lilium pumilum.*

**Results:**

Ultrastructural changes reflected dormancy release during cold storage of the bulbs. We compared gene expression levels among samples at 0 (S1 stage), 30 (S2 stage), 60 (S3 stage) and 90 (S4 stage) d of cold storage, with 0 d as the control. The data showed that some regulatory pathways such as carbohydrate metabolism and plant hormone signal transduction were activated to break dormancy. Some differentially expressed genes (DEGs) related to antioxidant activity, epigenetic modification and transcription factors were induced to respond to low temperature conditions. These genes constituted a complex regulatory mechanism of dormancy release.

**Conclusions:**

Cytological data related to dormancy regulation was obtained through histomorphological observation; transcriptome sequencing provided comprehensive sequences and digital gene expression tag profiling (DGE) data, and bulb cell ultrastructural changes were closely related to DEGs. The novel *Lilium pumilum* genetic information from this study provides a reference for the regulation of dormancy by genetic engineering using molecular biology tools.

**Electronic supplementary material:**

The online version of this article (10.1186/s12864-018-4536-x) contains supplementary material, which is available to authorized users.

## Background

The dormancy of bulbs is influenced by many factors. The inhibition and induction model of hormone-mediated regulation involves hormone-mediated inhibition of bud growth and anabolism, stimulation of shoot meristem growth, starch degradation and carbohydrate metabolism [1]. Abscisic acid (ABA) and gibberellin (GA) are important regulators of plant dormancy and germination, and they mitigate biotic and abiotic stress [2, 3]. Ethylene (ETH) is a hormone that affects growth and development from germination to senescence [4]. Previous studies show that ETH breaks dormancy by antagonizing ABA and promotes radicle processes [5]. Carbohydrate metabolism provides energy for starch hydrolysis and soluble sugar synthesis, which contributes to dormancy release. Energy metabolism also participates in dormancy regulation [6].

It has been found that reactive oxygen species (ROS) regulate both dormancy release and signal transduction [7, 8]. Many stress responsive genes (the transcription factors *WRKY*, *CBF*, *MYB* and *bZIP*) are also involved in dormancy regulation [9–11]. Sequencing technology is a scientific means of exploring the molecular mechanisms of plant dormancy, and has been widely used in this field of research. Examples include transcriptome analyses of Japanese apricot buds using 454-pyrosequencing technology [12], Japanese pear [13], and Chinese white pear ‘Suli’ flower buds [14].

Low temperature is an important environmental factor in bulb dormancy release and bud germination. During dormancy release, upstream and downstream factors regulate the transcription of many genes. Low temperatures influence the upstream regulation of GA biosynthesis, enhancing the biological activity of GA and thereby promoting dormancy release [15]. However, due to interspecific differences, dormancy types and treatment methods, there may be numerous differences in the effect of low temperature on dormancy. Dormancy release is related to the length of dormancy and the bulb storage method, and longer treatment times promote rapid germination [16].

*Lilium pumilum* originated from northern China and is an important, widely distributed, ornamental plant with rich nutritional value [17]. *L. pumilum* is highly resistant to Fusarium, leaf blight, drought, salinization and cold and is important for *Lilium* resistance breeding. In this study, we examined the ultrastructure and starch granules of bulb cells during cold storage. Cell morphology changes reflected the dormancy release process. Based on the cytological and physiological studies, a large number of dormancy-related genes were identified by transcriptome sequencing. This study enriches the genome information of *L. pumilum*, and provides valuable insights into dormancy release during cold storage of *Lilium pumilum*.

## Results

### Ultrastructure of bulb cells during cold storage

**Fig. 1:**
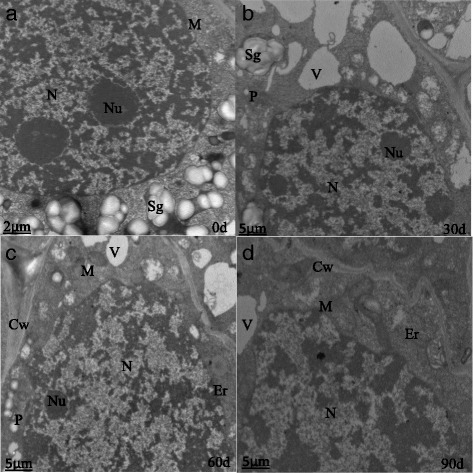
The nuclear ultrastructure in the apical bud of *Lilium pumilum* during cold storage. **a, b, c** and **d** represent the nuclear ultrastructure of the apical bud at 0 d, 30 d, 60 d and 90 d of cold storage, respectively. Cw, Cell wall; Er, Endoplasmic reticulum; M, Mitochondrion; N, Cell nucleus; Nu, Nuclei; P, Plastid; Sg, Starch granule; V, Vacuole

**Fig. 2:**
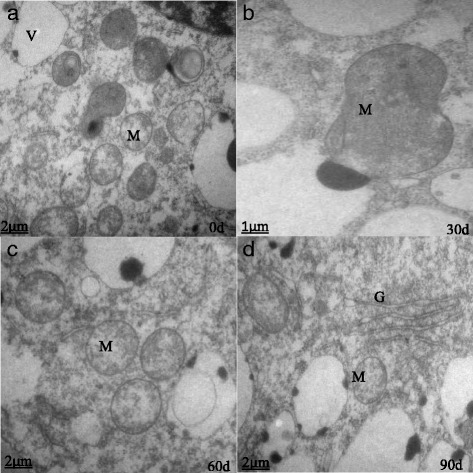
The mitochondria ultrastructure in the apical bud of *Lilium pumilum* during cold storage. **a**, **b**, **c** and **d** represent mitochondria ultrastructure in the apical bud at 0 d, 30 d, 60 d and 90 d of cold storage, respectively. G, Golgi body; M, Mitochondrion; V, Vacuole

**Fig. 3:**
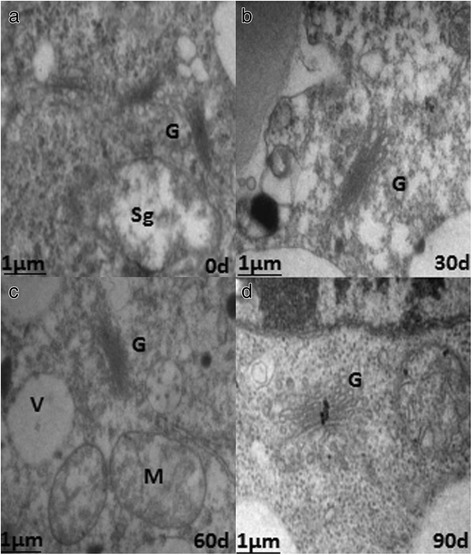
Ultrastructure of Golgi bodies in the apical bud of *Lilium pumilum* during cold storage. **a**, **b**, **c** and **d** represent ultrastructure of Golgi bodies in the apical bud at 0 d, 30 d, 60 d and 90 d of cold storage, respectively. G, Golgi body

Figure 6 contains small text. Please provide replacement figure file. Otherwise, please confirm if we can retain the current presentation.The nuclear volumes did not change significantly after 60 d of cold storage, but the nucleoli became compacted and clear (Fig. 1a, b, c). After 90 d, the nuclei were larger, and the nucleoli were loose (Fig. 1d). At harvest (S1), bulb cells had abundant mitochondria with a small number of visible tubular bubble crests (Fig. 2a), reduced Golgi, and fewer secretory vesicles (Fig. 3a). After 30 d of cold storage, mitochondria became elongated (Fig. 2b), and Golgi bodies consisted of 3–5 layers of stacked flat vesicles (Fig. 3b). After 60 d of cold storage, many spherical mitochondria were observed (Fig. 2c), and Golgi vesicles of bulb cells were stacked up to 6–7 layers, and large vacuoles appeared near the nuclei (Fig. 3c). There were no significant changes in plastids from 0 to 60 d. After 90 d of cold storage, many spherical mitochondria were still visible (Fig. 2d); Golgi vesicles were stacked up to 8–10 layers (Fig. 3d); more small and large vacuoles and irregular shaped plastids appeared in the cells; and there was a marked increase in numbers of long linear endoplasmic reticula (Fig. 1d).

**Fig. 4:**
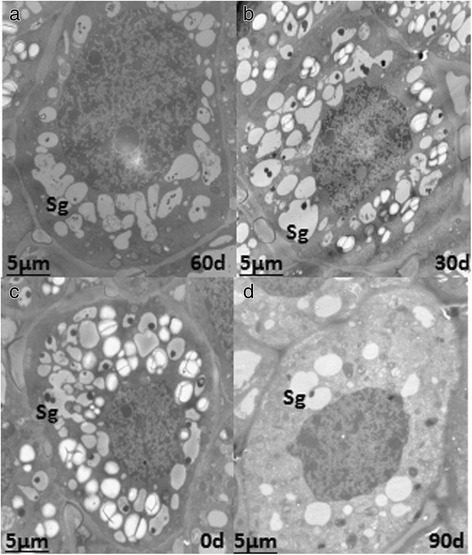
Starch granule changes in apical cells. **a**, **b**, **c** and **d** represent starch granules at 0 d, 30 d, 60 d and 90 d of cold storage, respectively.Sg, Starch granule

Parenchyma cells were completely filled with starch granules upon initial harvest (Fig. 4), which significantly decreased after 90 d of cold storage.

The numbers and types of organelles increased to regulate the intracellular environment and perform a variety of biological functions. Mitochondria are the main sites for cellular aerobic respiration, and they provide energy for dormancy release and regulate cell growth and the cycle during cold storage. The Golgi and endoplasmic reticulum constitute the important endometrial system.The degradation of starch granules provides a source of energy for various biological processes. The structure and function of the organelles are interdependent, and based on the ultrastructural changes, we infer that dormancy release occurs during cold storage.

### Transcriptome sequencing of bulbs during cold storage

#### Sequencing data

A total of 62.04 Gb of high quality data (four developmental stages and three biological replicates, the raw reads files were deposited in the NCBI Sequences Read Archive under SRP108930) was obtained after filtration, and after de novo assembly, the data were assembled with trinity to yield 189,271 transcripts and 80,193 unigenes. The N50 of the transcripts and the unigenes were 1540 and 1228, respectively, indicating high assembly quality. The unigene length distribution is shown in Additional file 1. Unigene sequences were compared and annotated with nr (ftp://ftp.ncbi.nih.gov/blast/db/), Swiss Prot (https://www.uniprot.org/), GO (http://www.geneontology.org/), COG (http://www.ncbi.nlm.nih.gov/COG/), KOG (ftp://ftp.ncbi.nih.gov/pub/COG/KOG/kyva), Pfam (http://pfam.xfam.org/) and KEGG (http://www.genome.jp/kegg/) databases (Additional file 2).

#### Analysis of DEGs

**Fig. 5:**
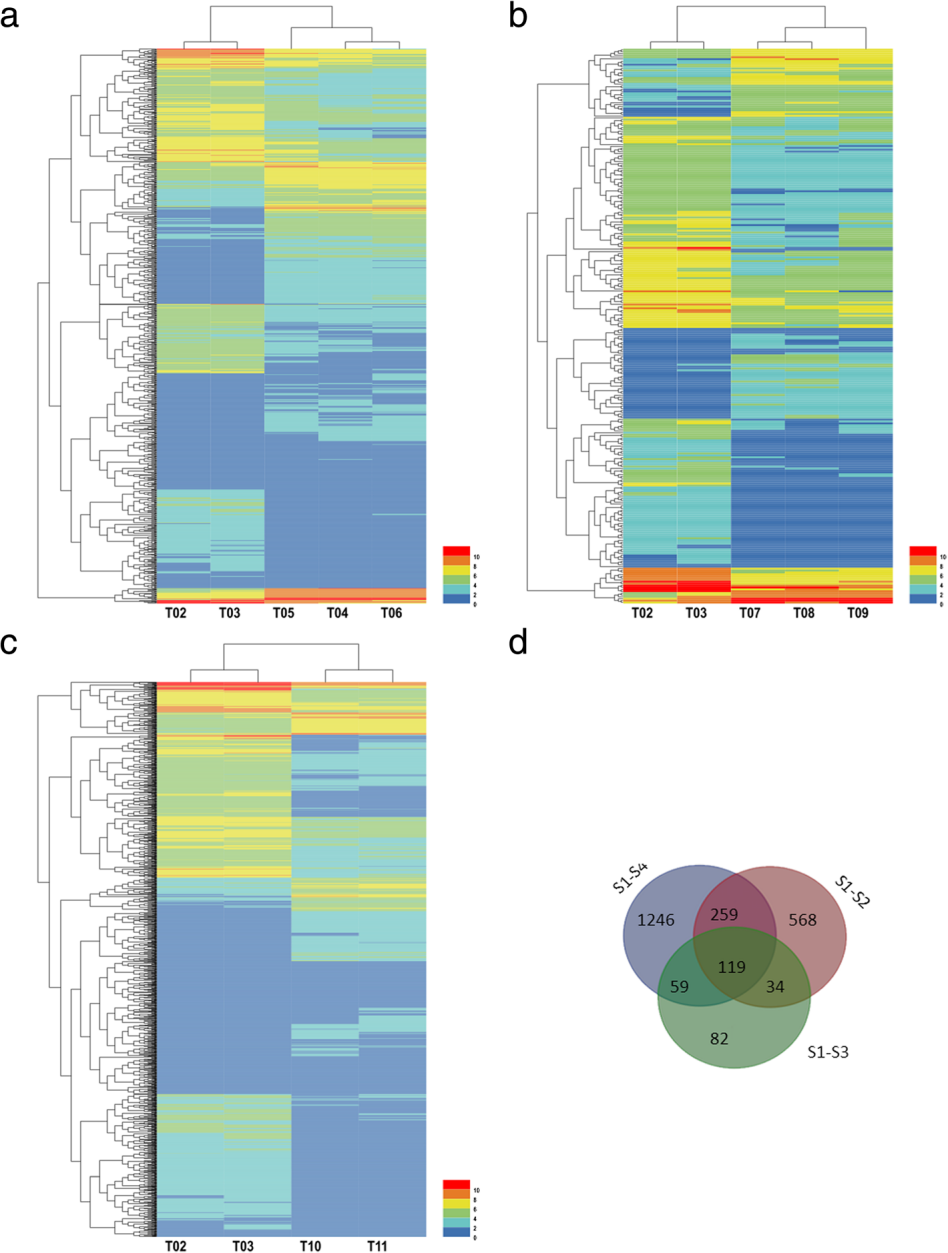
Hierarchical cluster and distribution of DEGs. **a**, **b** and **c** represent the cluster heat map in S1 vs S2, S1 vs S3 and S1 vs S4, respectively. **d** Venn diagram of DEGs

DEGs were significantly enriched during cold storage (Table 1). There were more DEGs in S1 vs S4 relative to S1 vs S2 and S1 vs S3, suggesting a greater complexity for the regulation of dormancy in S4. The hierarchical cluster and distribution of DEGs are shown in Fig. 5.

**Table 1:** The number of DEGs

DEGs Set	All DEGs	up-regulated	down-regulated
S1 vs S2	980	477	503
S1 vs S3	294	90	204
S1 vs S4	1683	726	957

#### GO enrichment analysis of DEGs

**Fig. 6:**
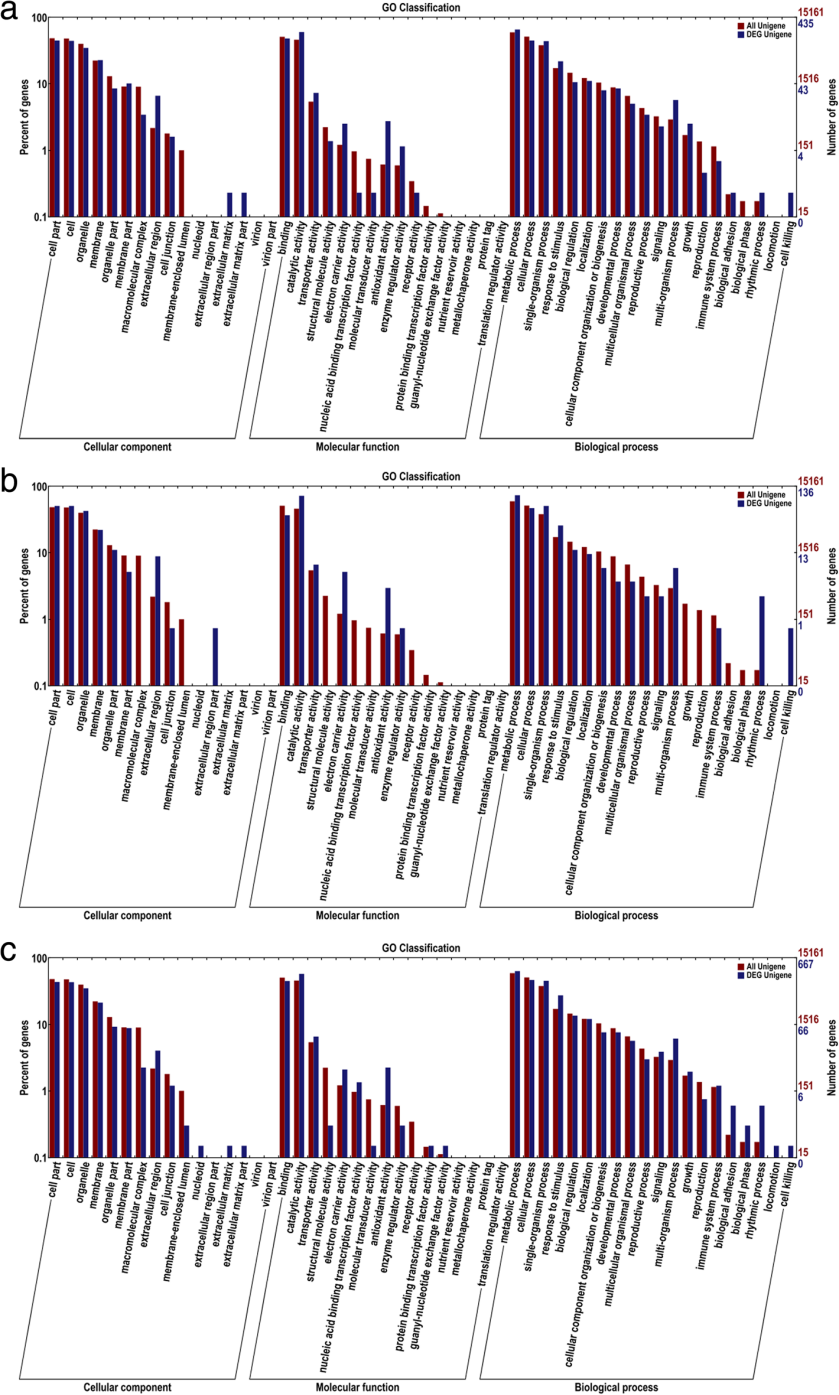
GO enrichment diagrams. **a, b** and **c** represent the secondary GO term annotation for DEGs in S1 vs S2, S1 vs S3 and S1 vs S4, respectively

There were 65,892 unigenes that were annotated in the GO database. These unigenes were divided into three subcategories: biological process, accounting for 45.6%; cellular component, accounting for 22.4%; and molecular function, accounting for 32%. There were 2,337 and 764 and 3,585 DEG unigenes in S1 vs S2, S1 vs S3 and S1 vs S4, respectively (Fig. 6, Additional file 3). Representative subclasses such as metabolic processes and catalytic activity, were significantly enriched, which implies that activation of energy metabolism pathways and related enzymes are required for dormancy release. Enrichment also occurred for genes associated with cellular processes, such as cell, cell part and cellular process. Many cells divide and grow after dormancy release, and the number and volume of cells increase during vegetative growth.

#### KEGG enrichment analysis of DEGs

**Fig. 7:**
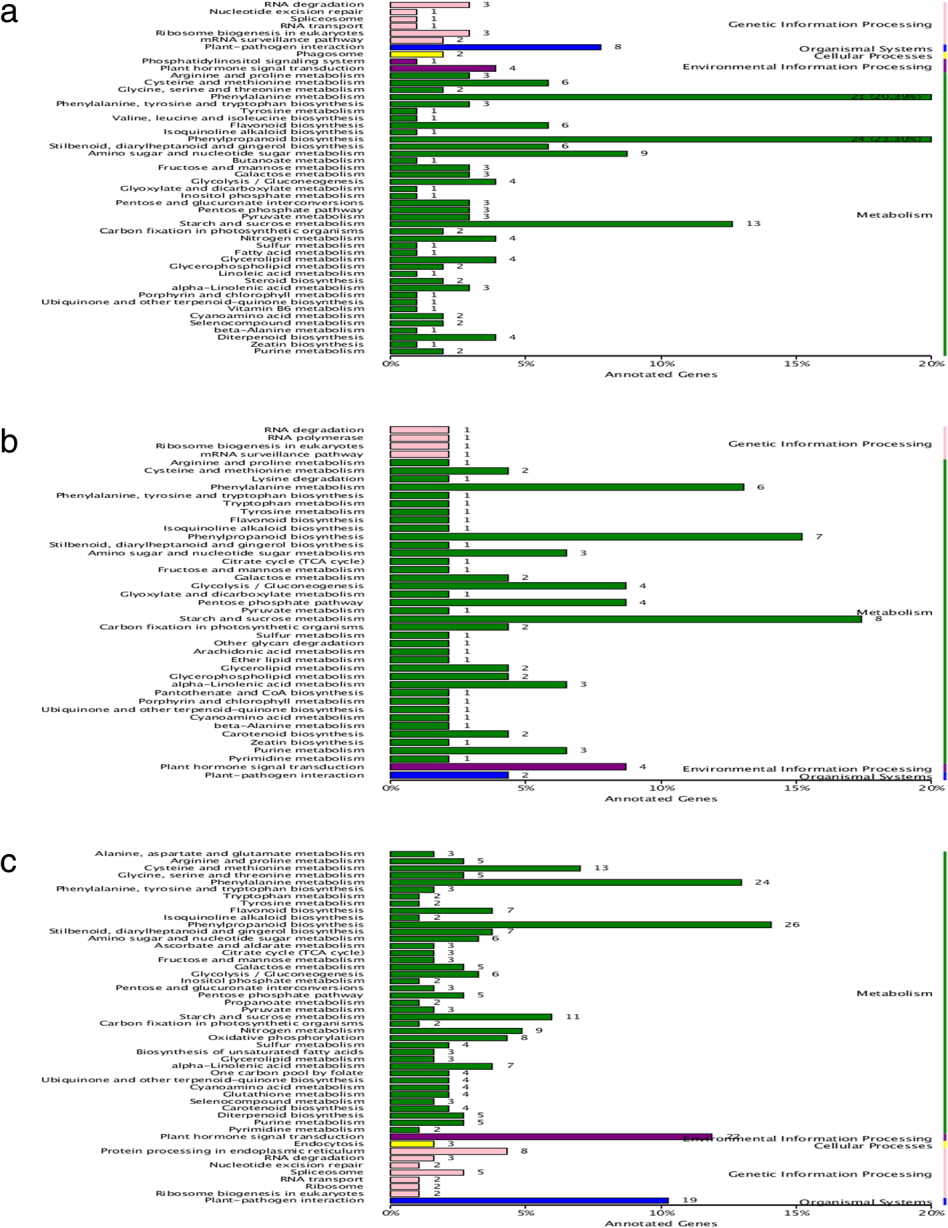
KEGG enrichment diagrams. **a, b** and **c** represent the KEGG categories for genes in S1 vs S2, S1 vs S3 and S1 vs S4, respectively

In total, 5596 genes including 119 pathways were annotated in the KEGG database, of which ribosome (ko03010), ribosome biogenesis in eukaryotes (ko03008), RNA degradation (ko03018), spliceosome (ko03040) and RNA transport (ko03013) annotated the most genes.The DEG unigenes in S1 vs S2, S1 vs S3 and S1 vs S4 were involved in 64, 43 and 80 pathways, respectively (Fig. 7, Additional file 4). We found some representative secondary metabolic pathways, such as phenylpropanoid biosynthesis and flavonoid biosynthesis. Genes encoding enzymes in biosynthetic pathways, such as flavonol synthase, chalcone synthase and chalcone isomerase were down-regulated during cold storage. Secondary metabolites are defensive compounds and may consume plant nutrients during their accumulation. The down-regulation of secondary metabolite-related genes may be a self-protection strategy against various abiotic stresses. Starch and sucrose metabolism as well as plant hormone signal transduction were also enriched. The former regulates plant energy metabolism, while the latter regulates endogenous hormone synthesis. These metabolic pathways interact to constitute a complex dormancy regulation network.

#### DEGs involved in starch and sucrose metabolism pathway during dormancy release

**Fig. 8:**
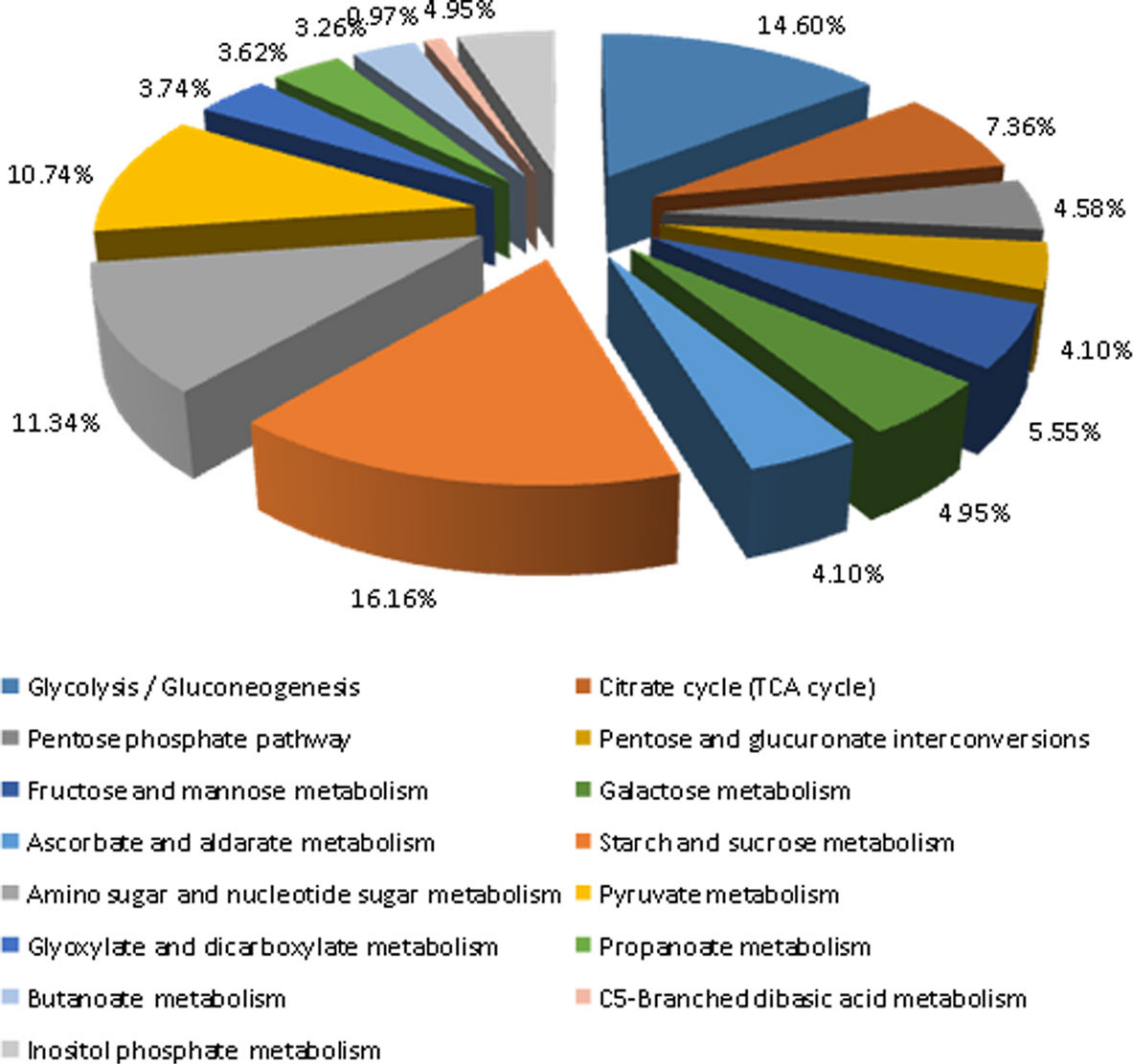
The unigenes distribution profiles involved in carbohydrate metabolism pathways

Cold acclimation is a complex process that involves a wide range of plant metabolic groups. Carbohydrate metabolism is an important component of the reprogrammed metabolic group especially under low temperature conditions, and the conversion of stored starch into soluble carbohydrates during cold acclimation has been widely reported in various plant species [18]. There were 829 unigenes involved in carbohydrate metabolism pathways, including starch and sucrose metabolism, glycolysis/gluconeogenesis, amino sugar and nucleotide sugar metabolism, and pyruvate metabolism (Fig. 8), of which, starch and sucrose metabolism accounted for the largest proportion (134/829). We focus on the differential expression of α-amylase (AMY) and β-amylase (BMY) related genes, only *c111346.graph_c1*(AMY) and *c114414.graph_c0* (BMY) were expressed in S1 vs S2; most of the DEGs were enriched in S1 vs S4 and many were up-regulated (Table 2, Additional file 5). Meanwhile, we found that the DEGs involved in sucrose synthesis were also up-regulated, including *c93866.graph_c0* and *c94446.graph_c0*. During the dormancy, the carbohydrates were mainly present in the form of starch, and at the later stage of the low temperature storage, starch hydrolysis accompanied by accumulation of sucrose released the metabolic energy for bulb dormancy release [19].

**Table 2:** The DEG unigenes involved in dormancy release pathways

DEG Unigene	S1 vs S2		S1 vs S3		S1 vs S4	
	up	down	up	down	up	down
Starch and sucrose metabolism						
Starch degradation	0	100%	67%	33%	57%	43%
Sucrose synthesis	0	0	100%	0	100%	0
Energy Metabolism						
Glycolysis /Gluconeogenesis	0	100%	33%	67%	33%	67%
PPP	33%	67%	33%	67%	60%	40%
TCA cycle	0	100%	100%	0	67%	33%
Plant hormone signal transduction						
ABA	0	100%	67%	33%	0	100%
GA	0	100%	0	0	0	100%
ETH	0	0	0	0	100%	0
JA	0	100%	0	0	0	100%
SA	0	100%	0	0	0	100%

#### DEGs involved in energy metabolism pathway during dormancy release

There were 121, 61 and 38 unigenes involved in glycolysis/gluconeogenesis, the citrate cycle (TCA cycle) and the pentose phosphate pathway (PPP), respectively, which are the three main pathways for plant energy metabolism. The DEG encoding pyruvate dehydrogenase E1 (*c110227.graph_c0*), which is responsible for entry of pyruvate into the TCA cycle, was only expressed in S1 vs S2 (Table 2, Additional file 5); perhaps it is mainly involved in the transition from S1 to S2 stage. The other enzymes involved in subsequent steps, including 2-oxoglutarate dehydrogenase (*c110805.graph_c0*) and isocitrate dehydrogenase (*c89446.graph_c*), were up-regulated in S1 vs S4, and the TCA cycle may be activated in the later stage of cold storage. The key enzymes encode the genes ATP-dependent 6-phosphofructokinase 3 (*c97761.graph_c0*), ATP-dependent 6-phosphofructokinase 2 (*c101812.graph_c0*), 6-phosphogluconolactonase 4 (*c102778.graph_c0*) and ribose-5-phosphate isomerase (*c95301.graph_c0*), which are involved in PPP, and the up - regulation of the DEGs indicated that the PPP is induced by cold treatment. In glycolysis/gluconeogenesis, some of the DEGs were up-regulated, including ATP-dependent 6-phosphofructokinase 3 (*c97761.graph_c0*) and 2,3-bisphosphoglycerate-independent phosphoglycerate mutase (*c76005.graph_c0*). However, the down- regulated DEGs accounted for a greater proportion.

#### DEGs involved in hormone signal transduction pathway during dormancy release

A variety of plant hormones interact to constitute a complex dormancy regulation network. ABA is necessary to induce plant dormancy and inhibit germination, while GA promotes germination [20]. The carotenoid metabolism pathway was associated with ABA metabolism, and 9-cis-epoxycarotenoid dioxygenase (NCED) and carotenoid cleavage dioxygenase 4 (CCD) regulated ABA synthesis. Many of the DEGs were mainly enriched in S1 vs S4 and all of them were down-regulated (Table 2, Additional file 5). Correspondingly, the DEGs encoding abscisic acid 8&apos;-hydroxylase 2 (CYP707A), which are involved in ABA degradation, were all up-regulated in S1 vs S3. ABA concentrations may be reduced during low temperature storage. The diterpenoid biosynthesis pathway was associated with GA metabolism. Gibberellin 2-beta-dioxygenase(GA2ox1) and gibberellin 3-beta-dioxygenase (GA3ox4) were related to five down-regulated DEGs that inactivate GA; however, none of the DEGs encoding gibberellin 20 oxidase (GA20ox) that participated in GA synthesis were found. We further analysed the data and found that there were six genes encoding GA20ox (not listed); these genes regulate GA synthesis, but the roles did not show a difference in different stages of cryogenic storage. The ACC oxidase transcript (*c97695.graph_c0*), which oxidizes ACC to ETH, was only expressed in S1 vs S4 and up-regulated, thereby increasing the ETH levels. Alpha-linolenic acid metabolism and phenylalanine biosynthesis are related to the jasmonic acid (JA) and salicylic acid (SA) biosynthesis pathways. However, the DEGs encoding linoleate 13S–lipoxygenase and phenylalanine ammonia-lyase, which are involved in JA and SA synthesis, respectively, were enriched in S1 vs S2 and S1 vs S4, all of them were down- regulated. The finding was consistent with the study of Glenn et al. [21].

#### DEGs involved in antioxidant reactions during dormancy release

The equilibrium between oxidation and the antioxidant system is shifted by plant cell respiration and energy metabolism, which promotes the accumulation of ROS and leads to oxidative stress. When the concentration of ROS is sufficient, bulb dormancy can be relieved [22]. Several key enzymes were found to be involved in the antioxidant reaction, such as catalase (CAT), alcohol dehydrogenase (ADH), stilbene synthase (StSy) and glutathione S-transferase (GST) (Table 3, Additional file 6). Our data showed that the transcript of CAT (*c104193.graph_c1*) was reduced in S1 vs S4. The down-regulation of CAT could increase the hydrogen peroxide content to trigger dormancy release [23]. The DEGs encoding ADH were only enriched in S1 vs S4, of which, *c106934.graph_c0* was up-regulated. The induction of the ADH gene could reduce the oxidative phosphorylation pathway induced by cold treatment and artificial stimuli [24]. Antioxidative machinery such as StSy and GST were significantly enriched in S1 vs S4; many of these genes were induced, which indicated that the ascorbate-glutathione cycle was probably induced to cope with the increased oxidative stress in the bud at the later stage of low temperature storage [6].

**Table 3:** The DEG unigenes involved in cellular activity

DEG Unigene	S1 vs S2		S1 vs S3		S1 vs S4	
	up	down	up	down	up	down
Antioxidant reaction	100%	0	100%	0	42%	58%
DNA methylation	0	0	0	0	0	100%
Cell division and cell growth	67%	33%	0	0	86%	14%

#### DEGs involved in DNA methylation during dormancy release

DNA methylation increased throughout the genome in an ABA-dependent manner [25]. However, DNA de-methylation occurred prior to transcriptional activation of genes involved in cell division and meristematic growth [26]. We focused on the biological process related to DNA methylation (GO:0006306) and DNA demethylation (GO:0080111), in total, 68 and 1 genes were annotated, respectively. Of which, two DEGs related to DNA (cytosine-5)-methyltransferase were down-regulated in S1 vs S4 (Table 3, Additional file 6). Low temperature affects epigenetic modification through DNA methylation [27]. Transcriptional silencing triggered by DNA methylation may lead to growth arrest during dormancy; the reduction of DNA methylation may respond to dormancy release at the later stage of low temperature storage. However, the genes involved in DNA demethylation were not differentially expressed.

#### DEGs involved in cell division and growth during dormancy release

Plant growth and development remain stagnant during dormancy, while cell division is accelerated to meet the needs of vegetative growth after dormancy release [28]. There were 53 and 39 genes that participated in cell division (GO:0051301) and cell growth (GO:0016049) biological processes. All of the DEGs were enriched in S1 vs S4 and many of them were up-regulated (Table 3, Additional file 6). Meanwhile, cyclin-A and cyclin-D, were closely related to the mitotic activity of the shoot meristem [29]. In our study, *c77281.graph_c0* encoded cyclin-A and *c104987.graph_c0* encoded cyclin-D, both of them were up-regulated to stimulate mitotic activity in response to the dormancy release. Cell mitosis is also the cause of the increase in the number and volume of cells to meet the need for vegetative growth after bulb dormancy release.

#### QRT-PCR validation of DEGs

**Fig. 9:**
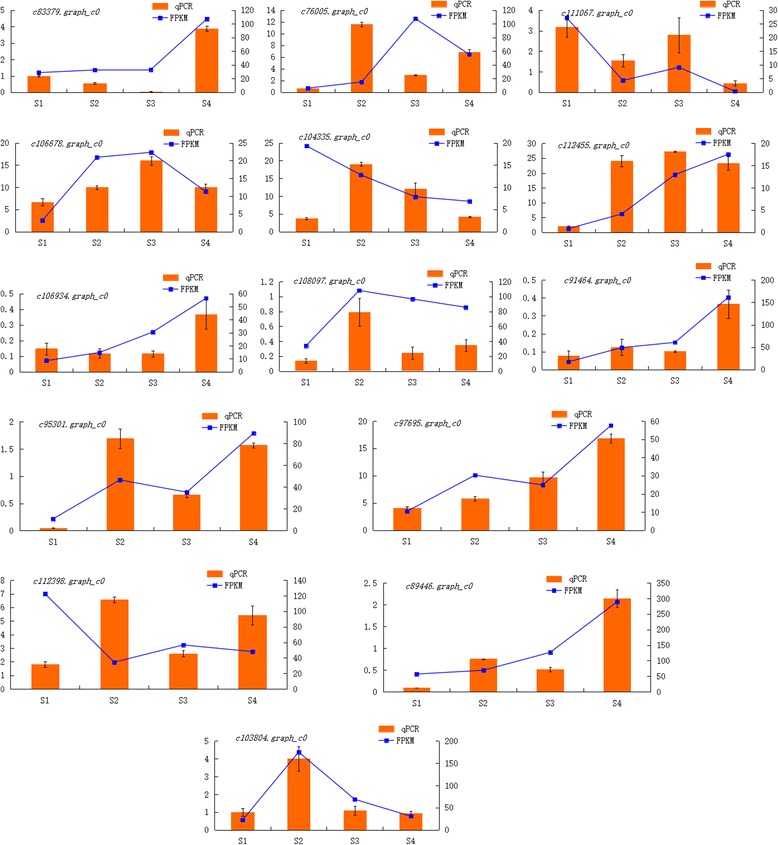
QRT-PCR validation of DEGs

To verify the results of the transcriptome data, 14 DEGs were randomly selected for qRT-PCR analysis (Fig. 9), the primer information was shown in Additional file 7. Most of the transcriptome data agreed with the qRT-PCR results; the few inconsistencies may have been due to the differences in the sensitivity, specificity, and the algorithms for qRT-PCR and sequencing technology.

#### Transcription factors responding to abiotic stimulus

**Fig. 10:**
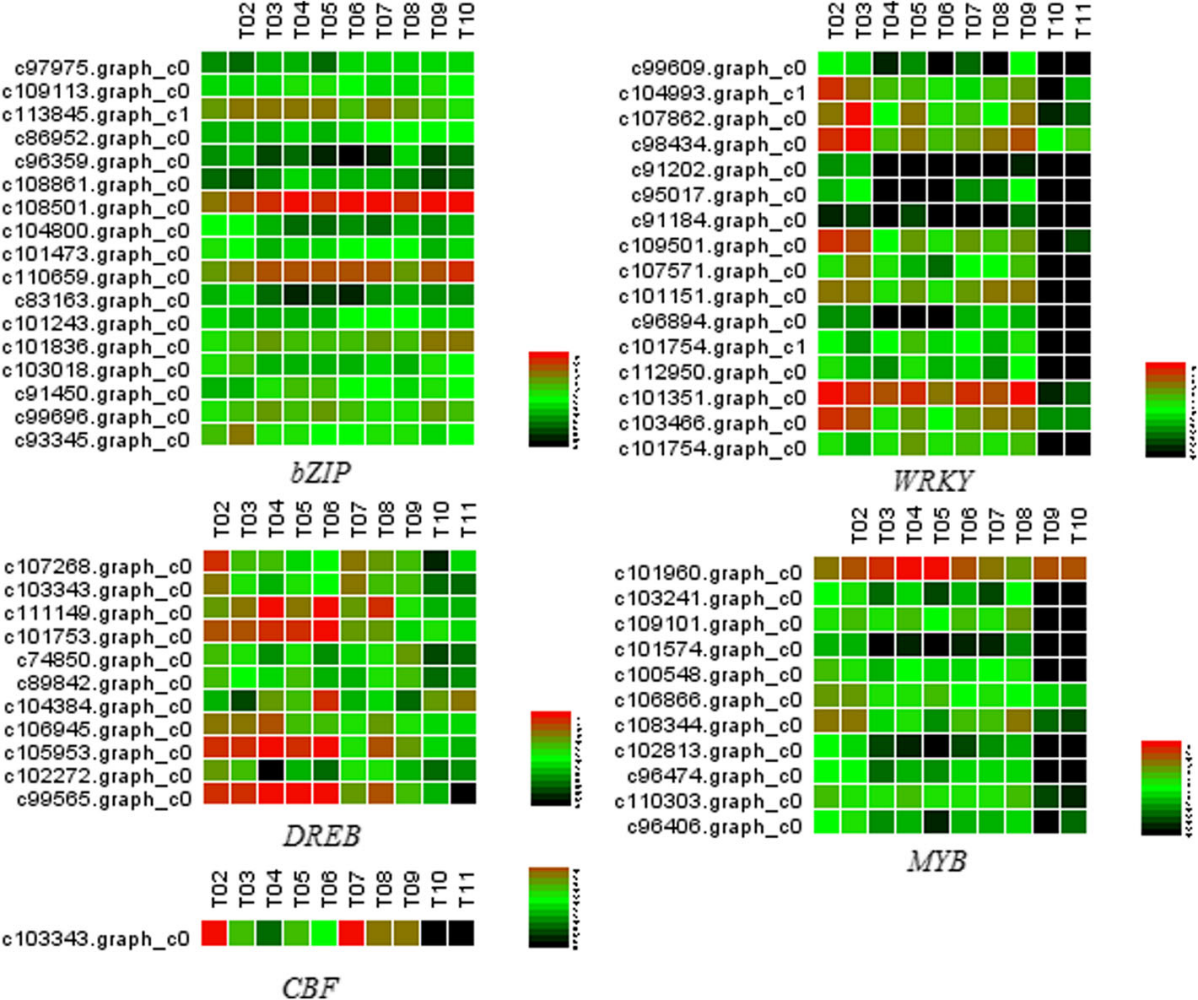
The heat map of related transcription factors

The cold acclimation process is accompanied by a variety of abiotic stimuli. Cold temperatures trigger the expression of the *CBF* family of transcription factors, which in turn activate many downstream genes that confer freezing tolerance to plants [30]. However, *CBF* was not enriched, and only one was found during cold storage (Fig. 10). Bai et al. [13] observed the same results with a transcriptome analysis of Japanese pear flower buds transitioning through endodormancy. The result is beyond our expectation. Although the important role of low temperature in dormancy release is widely recognized, how the frozen signal is sensed and transduced to the downstream factors has been in the discussion. In *Arabidopsis*, *MYB* participates in cold stress, regulates ABA-responsive genes and plays an important role in the upstream step of cold stress signalling transduction [31]. The *WRKY* gene family plays important roles in abiotic stress tolerance and ABA- signalling [32]. *WRKY* is known to promote the expression of anti-freezing protein (AFP) in response to abiotic stress [33]. *MYB* and *WRKY* were up-regulated in the early cryopreservation (Fig. 10), and they act as potential upstream regulators in freezing or may be involved in tolerance to multiple stresses. In addition, *bZIP* and *DREB* also regulate the expression of many stress-inducible genes, mostly in an ABA-independent manner, and play key roles in improving plant abiotic stress tolerance [34, 35]. Our data showed that *bZIP* and *DREB* were significantly enriched (Fig. 10).

## Discussion

Low temperature is an important environmental signal for dormancy release. We identified a large number of DEGs comprising a complex network of dormancy regulation. The study provides insight into the mechanism of *L. pumilum* dormancy and has added greatly to the molecular data for bulbous plants.

### Changes in bulb cell ultrastructure during low temperature storage

The plant cell ultrastructural changes are closely related to the process of dormancy release. A previous study found that in the seasonal dormancy buds of poplar, the cell wall thickens, the storage of protein in the vacuole increases, and contraction and blocking of the intercellular connection leads to interruption of the transport of the comonomer, limiting the exchange and signal transduction between adjacent cells, which in turn leads to growth arrest and dormancy [36]. And increase in the number of organelles, such as mitochondria, plastids, Golgi bodies, vacuoles and micro bodies, in the apical meristem cells occurs when dormancy is broken [37]. Cold treatment relieves the inhibition of specific transcription and translation sequences, leading to structural changes in stem cells [38]. With the prolongation of low temperature storage, the nuclei volume increased. The nucleus plays an important role in metabolism, growth and differentiation of cells and is the main site of genetic material [39]. The type and number of organelles were constantly enriched, including endoplasmic reticulum, mitochondrion, plastids, vacuoles and Golgi bodies. Mitochondria are the main place for aerobic respiration, and the energy released satisfies that needed for dormancy release [40]. Golgi bodies constitute the inner membrane system of cells that play an important role in material transport. The increase in the layers of Golgi vesicles and the complexity of the structure showed that intracellular vesicle trafficking plays a role in the regulation and execution of bulb dormancy release [41]. As an important storage form of carbohydrates in lily bulbs, starch is often present in the form of starch granules. The number of starch granules in bulb cells decreased gradually during low temperature storage, which indicated that starch is continuously degraded in response to dormancy release.

### Changes in gene expression levels during low temperature storage

#### The key regulatory pathways were in response to dormancy release

A shift in the relationship between sugar, starch and soluble sugar was found in studies of lily cryopreservation. The soluble sugar content was used as a judge to break dormancy and acts as a signaling molecule to regulate the growth of buds [42, 43]. Therefore, such starch-sugar interconversions might be mediated, in part, by the co-action of AMY, BMY and sucrose synthase (SuSy). The up-regulated DEGs related to AMY and BMY indicated that starch is degraded to provide metabolic energy for dormancy release and subsequent vegetative growth. In many studies, the increase in soluble sugar content is highly correlated with the enhancement in cold tolerance [44]. The DEGs related to SuSy were up-regulated in response to cold, water deficit, hypoxia and salt stress. Furthermore, SuSy simulated hypoxic conditions and cues in mitochondria together with pyruvate decarboxylase and alcohol dehydrogenase, to participate in the regulation of oxidative stress and dormancy release in plants [45]. Therefore, sucrose acts as an induction signal and is involved in not only dormancy release but also the formation of the protection mechanism under stress conditions.

The transition from dormancy to bud growth needs to consume energy. In grapes, Ophir et al. [46] suggested that HC- and heat shock (HS)-induced dormancy release involves the TCA cycle and ATP synthesis. Gai et al. [6] found several key enzymes that participated in the TCA cycle were up-regulated during artificial chilling induced dormancy release of *Paeonia ostii*. *c110805.graph_c0* and *c89446.graph_c0*, encoding the main enzymes, 2-oxoglutarate dehydrogenase and isocitrate dehydrogenase, respectively, were up-regulated in S1 vs S4, which indicated that the TCA cycle is induced. Our finding was consistent with the enzyme activities during chilling induced dormancy release in *Curcuma longa* and *Dioscorea esculenta* [47]. Dormancy release is a biological process of energy consumption. The energy released by aerobic metabolism meets the need for dormancy release, and metabolism produces a variety of intermediates that provide compound carbon framework for the growth of cells after dormancy release [6, 47]. The PPP was also activated at the later stage of low temperature storage. Previous studies have found that PPP activation is associated with dormancy release both in perennial buds and seeds [24]. In addition, the defence response produced by plants under low temperature conditions can also induce the PPP, which provide antioxidant protection against ROS by producing NADPH [47]. The activation of the PPP is not only a signal of dormancy release, but also a protective mechanism under plant stress conditions. However, the down-regulated DEGs that were involved in glycolysis/gluconeogenesis accounted for more proportion. Glycolysis / gluconeogenesis may be inhibited. These results were not consistent with the response of *Paeonia ostii* to artificial cold treatment [6], but agreed with the studies of grapes and other dormant plants [46, 48].

Crosstalk among plant hormones coordinates the regulation of flowering and fruiting, maturation and senescence, as well as the dormancy and germination processes. The balance between ABA and GA determines plant dormancy [20]. The ABA/GA balance is disturbed when the plant undergoes environmental changes; low temperature conditions were associated with low ABA biosynthesis, which has an antagonistic effect to the GA response in numerous physiological processes [49] .Our data showed that the DEGs related to ABA synthesis, including NCED and CCD, were enriched in S1 vs S4 and were down-regulated. Meanwhile, GA2ox1 and GA3ox4, which are involved in GA degradation, were also down-regulated. GA may dominate to promote bulb dormancy release under low temperature conditions. ETH actively regulates dormancy release by reducing ABA synthesis, enhancing ABA degradation, or changing ABA activity [50]. Ophir et al. [46] found that the DEGs related to ETH biosynthesis and signal transduction were up-regulated during grape bud dormancy release. In our study, *c97695.graph_c0* encoding AC0, was up-regulated to increase the ETH synthesis level, which agreed with previous studies. It was reported that SA promoted dormancy release by measuring H_2_O_2_ production in grapes [51]. However, Nugroho et al. [52] noted that the differential expression associated with SA genes was not caused by plant dormancy but be more closely related to anthocyanins in the phenylpropanoid pathway and changes in flavonoid biosynthesis. In *Arabidopsis* mutants, germination rates correlate strongly with elevated JA levels [53]. Our data showed that the levels of JA and SA were higher in the early stage of low temperature storage. Increases in JA and SA concentrations were associated with plant responses to stress and the development of the vegetative axis [54].There is no clear consensus for the influence of JA / SA on dormancy regulation.

#### The genes were differentially expressed to adapt to low temperature conditions

Dormancy release induction by natural or artificial stress signals (such as low temperature) was associated with the antioxidation reaction [55]. ROS interacted with ABA and GA transduction pathway and were likely to control numerous transcription factors and properties of specific proteins through their carbonylation [56]. CAT is responsible for the removal of excess ROS during stress. It was reported that CAT inhibition accelerated dormancy release and sprouting in potatoes [57]. In our study, *c104193.graph_c1*, a gene encoding CAT, was down-regulated in S1 vs S4. CAT inhibition could result in an increase in the level of endogenous H_2_O_2_ in bulb tissues, which might activate PPP and thus lead to dormancy release and initiation of sprouting [58]. In addition, several main enzymes that were involved in the antioxidant reaction, including ADH, StSy and GST, were up-regulated at the later stage of low temperature storage, which was similar to antioxidant enzyme changes of *Paeonia ostii* during artificial chilling induced dormancy release [6]. These results indicate that bulb dormancy release induced by cold acclimation is associated with the removal of free radicals through activated peroxide-scavenging systems.

The plant responds to internal or external environmental signals through epigenetic modifications, including DNA methylation [59]. Previous studies have reported that the low temperature associated changes in DNA methylation are evident [60]. Kumar et al. [27] found that cold acclimation during dormancy in the apple was likely to affect the epigenetic regulation through DNA methylation. Our data showed that 68 genes were involved in DNA methylation; therefore, bulbs could modulate growth and development to adapt under cold stress with epigenetic mechanisms. The DEGs encoding DNA (cytosine-5)-methyltransferase were down-regulated in S1 vs S4. This finding was consistent with the analysis of Bai et al. [13]. With prolonged cold storage, the level of methylation decreased, which suggested that epigenetic regulation participates in bulb dormancy release.

### The regulatory mechanism of dormancy release during low temperature storage

#### The correlation between changes in bulb cell ultrastructures and gene expression levels

**Fig. 11:**
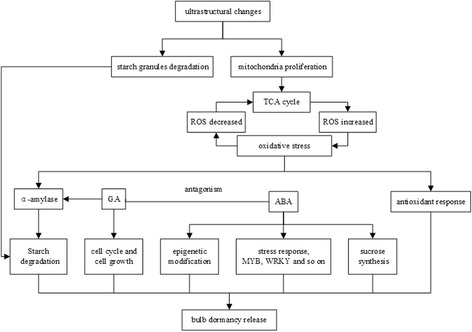
The regulatory mechanism of dormancy release during low temperature storage of *Lilium pumilum* bulbs

From ultrastructural observation, we found that the shape of mitochondria changed from a long stick to a sphere during low temperature storage. Mitochondria may divide to increase the abundance in number. Mitochondria are the main sites for aerobic respiration, which provides energy to meet the needs of dormancy release. Meanwhile, mitochondria are also important sources of ROS production [61]. With increased levels of ROS, many antioxidant enzymes such as CAT, ADH, StSy and GST, are activated. Therefore, peroxide-scavenging systems occurs [6]. In turn, the transcription and metabolite levels in the TCA cycle are reduced with the increased levels of ROS [46], thereby maintaining proper ROS concentrations and promoting dormancy release (Fig. 11).

In plants, sugar (or carbohydrate compounds) and phenolic compounds act as part of the redox system, removing ROS and contributing to stress tolerance [62]. Transient oxidative stress and respiratory stress may activates AMY, accelerating the hydrolysis of starch [63]. In addition, the activation of GA biosynthesis and signalling pathway by artificial chilling could also induce AMY gene expression [64]. Ultrastructural observation revealed that parenchyma cells were completely filled with starch granules at the initial time of low temperature storage, which significantly decreased in number after 90 d of cold storage. Correspondingly, the DEGs related to AMY were activated. The morphological changes of cells and the differential expression of related genes were consistent, which also suggested that starch hydrolysis coupled with the antioxidant reaction and GA signalling pathway was involved in bulb dormancy release (Fig. 11).

#### The regulatory network constituted by DEGs

The antagonism of ABA and GA determines plants dormancy [20]. It was reported that the expression of SuSy gene can respond to changes in endogenous ABA at transcription and post - transcriptional levels [65]. Sugar interacted with ABA signalling pathways in seeds [66]. Moreover, GA acted as a positive regulator of the cell cycle and cell growth, transforming the mitotic cycle to endocycle during cell differentiation, thereby affecting cell elongation [67]. It can be concluded that the biological processes including glucose metabolism, plant hormone signalling pathway, cell division and growth, are coupled with each other during bulb dormancy release (Fig. 11).

ABA is a broad-spectrum phytohormone involved not only in regulating growth and development but also in coordinating various stress signal transduction pathways when plants are under abiotic stress conditions [68]. The ABA signal transduction pathways from stress signal perception to gene expression involve different transcription factors, including *DREB*, *MYB*, *WRKY* and *bZIP*, during bulb cold storage. It has been reported that epigenetic processes are an integral part of ABA-regulated processes [69]. Bulb cells need to adapt to cold stress during low temperature storage, epigenetic modification and the differential expression of various transcription factors are in response to ABA signalling, therefore regulating dormancy release (Fig. 11).

## Conclusions

Morphological changes reflect the process of dormancy release in bulbs; transcriptome sequencing provides us with comprehensive sequences and DGE analysis data. This study enriches the genome information of *L. pumilum* and lays the foundation for exploring the mechanism of bulb dormancy.

## Methods

### Plant materials

The test materials were three-year-old bulbs of *L. pumilum*, with a circumference of 4–6 cm that were planted in the nursery of Northeast Forestry University and managed conventionally. The disease- and pest-free bulbs of *L. pumilum* were harvested in mid-October 2014. After 30 min of sterilization with 50% carbendazim WP 800 times aqueous solution, the samples were thoroughly washed and dried. Wet steam-sterilized perlite was used as the storage matrix, and the bulbs were placed in a refrigerator at 4°C for cold storage. Samples stored for 0, 30, 60, and 90 days were labelled as S1, S2, S3 and S4, respectively; they were used in the study of ultrastructure and transcriptome sequencing.

### Ultrastructure of bulb cells

Shoot apical meristem samples of bulbs were cut into 0.5–1 mm^3^ pieces and fixed in 2.5% glutaraldehyde fixative (vacuumed to submerge) at 4 °C for at least 24 h. The tissue materials were washed with 0.1 mol·L-1 phosphate buffer for 2 h, and then dehydrated with 30%, 50%, 70%, 80%, 90% and 100% ethanol; the dehydrated samples were infiltrated and embedded with Epon812. The samples were sliced with an ULTRACUTE ultrathin microtome and stained with uranyl acetate and lead citrate. The tissue sections were examined with an H-7650 transmission electron microscope. Three fields were selected and five cells in each field were examined for ultrastructural changes in the bulb cells and imaged.

### Total RNA isolation, library construction and transcriptome sequencing

Total RNA was extracted using the EASYspin Plus Plant RNA Rapid Extraction Kit (Cat. RN38). The quality of RNA was measured with a micro-spectrophotometer Nanodrop 2000 and agarose gel electrophoresis. RNA purity was measured by a NanoPhotometer® spectrophotometer (IMPLEN, CA, USA). RNA concentration was measured with the Qubit® RNA Assay Kit in Qubit® 2.0 Flurometer (Life Technologies, CA, USA). The concentration of RNA was 400 ng · μL^− 1^, and it had an A260:280 ratio of 1.8 to 2.0. The gel electrophoresis strips were clear with no tailing, and the brightness ratio of 28S to 18S was 2: 1. The RNA quality met sequencing requirements. The cDNA library was sequenced using the Illumina HiSeq 2500 high throughput sequencing platform, and a large number of high-quality reads were obtained. Most of the base quality scores met or exceeded Q30. Sequences were assembled withTrinity [70] to construct a sequencing library.

### Gene functional annotation and expression analysis

Unigene annotation information was obtained by comparing the unigene sequences to nr [71], Swiss-Prot [72], GO [73], COG [74], and KEGG [75] databases with BLAST [76] software (version 2.2.26). Eventually, 29,811 annotated unigenes were obtained.

The reads from each sequencing sample were compared to the unigene database with Bowtie [77]. The expression levels were estimated by RSEM [78] based on the alignment results. The expression levels of genes were based on FPKM [79], and the FPKM formula is as follows:

$$ FPKM=\frac{cDNA\kern0.34em Fragments}{Mapped\kern0.17em Fragments(Millions)\times Transcript\kern0.17em Length(kb)} $$

### Differential expression analysis, DEGs functional annotation and enrichment analysis

The Pearson correlation coefficient was used as an indicator of correlation between biological replicates [80], and the samples were highly correlated. The differences of expression between two groups were analysed by DESeq [81] and FDR (False Discovery Rate) < 0.01 and a fold change (the ratio of expression between two groups) ≥ 2 were used as the criteria to screen DEGs. Hierarchical cluster analysis was used to show the expression patterns of genes with the same or similar expression behaviour, and the DEGs were compared with GO, COG and KEGG databases for functional enrichment analysis.

### Validation by qRT-PCR

Fourteen DEGs were randomly selected for qRT-PCR. Primer information was shown in Additional file 1. Reactions were performed with Roche LightCycler 96: Preincubation (95 °C for 30s); three step amplification (95 °C for 5 s; 60 °C for 15 s; 72 °C for 30 s; 40 cycles); melting (95 °C for 10s; 65 °C for 60 s; 97 °C for 1 s); cooling (37 °C for 30 s). All experiments were done with three biological replicates. Relative expression was calculated according to the 2^-ΔΔCt^ algorithm, and was based on expression of the actin gene (JX826390).

## Additional files

Additional file 1: The unigene length distribution. (XLSX 9 kb)

Additional file 2: Statistics for de novo assembly of transcriptome. (XLSX 9 kb)

Additional file 3: GO enrichment analysis of DEGs. (XLS 33 kb)

Additional file 4: DEGs involved in KEGG pathways. (XLS 72 kb)

Additional file 5: The key regulatory pathways involved in dormancy release. (XLSX 15 kb)

Additional file 6: DEGs involved in cell activity during dormancy release. (XLSX 13 kb)

Additional file 7: qRT-PCR primer information. (XLS 24 kb)

## Abbreviations

ABA: Abscisic acid
DEGs: Differentially expressed genes
DGE: Digital Gene Expression Tag Profiling
ETH: Ethylene
GA: gibberellin
ROS: Reactive oxygen species
